# The Effect of Heparin on Bone Metabolism and Orthodontic Tooth Movement in Rats

**DOI:** 10.1002/cre2.70061

**Published:** 2025-03-11

**Authors:** Behzad Salari, Reza Moradian, Yasaman Kheirandish, Mojgan Alaeddini, Shahroo Etemad‐Moghadam, Shahla Maghsoudi, Ahmad Reza Dehpour

**Affiliations:** ^1^ Department of Orthodontics, Faculty of Dentistry, Tehran Medical Sciences Islamic Azad University Tehran Iran; ^2^ Student Research Committee, School of Dentistry Aja University of Medical Sciences Tehran Iran; ^3^ Department of Dentomaxillofacial Radiology, School of Dentistry Tehran University of Medical Sciences Tehran Iran; ^4^ Dental Research Center, Dentistry Research Institute Tehran University of Medical Sciences Tehran Iran; ^5^ Experimental Medicine Research Center Tehran University of Medical Sciences Tehran Iran; ^6^ Department of Pharmacology, School of Medicine Tehran University of Medical Sciences Tehran Iran

**Keywords:** bone density, heparin, orthodontic tooth movement, parathyroid hormone, rat

## Abstract

**Objectives:**

Various attempts have been made to increase the rate of orthodontic tooth movement (OTM). The aim of this study was to determine the effect of different doses of heparin on OTM and paraclinical factors related to bone metabolism in rats.

**Methods and Materials:**

A total of 24 Sprague‐Dawley rats were randomly divided into three groups of 8 animals each and injected with 0 (control), 3000, and 6000 U/Kg/d heparin sulfate for 4 weeks. Radiographs were obtained at the initiation and at the end of the study period. Orthodontic forces were applied on Day 14 and continued for the next 2 weeks, after which, OTM, optical density, parathyroid hormone (PTH) level, and histologic variables were assessed for each rat. The latter was performed on hematoxylin/eosin–stained sections of the mesial roots of the first molar and included calculation of the osteoclast number, and resorption lacunae depth and number. One‐way analysis of variance, the Tukey test, and a paired‐*t*‐test were used for statistical analysis (*p* < 0.05).

**Results:**

A significant increase in OTM, the number of resorptive lacunae, and PTH secretion was observed in the group that received 6000 U/Kg/d compared with both the other groups. There was no significant difference in optical density, and, therefore, bone density, among the study groups (*p* > 0.05).

**Conclusion:**

Heparin injection affects bone metabolism in rats, as shown by the increases in OTM and PTH and its impact on histologic parameters. These effects seem to be dose‐dependent and may be a factor that should be taken into consideration during orthodontic treatment planning.

## 1 Introduction

Orthodontic treatments are dependent upon tooth movement. Mechanical stimulants activate cellular responses in the periodontal ligament and alveolar bone, which enable the teeth to relocate by means of bone resorption and formation (Maltha and Kuijpers‐Jagtman 2023). The exact mechanism of orthodontic tooth movement (OTM) is unknown, but it is regarded as a relatively slow process, leading to long treatment durations (Maltha and Kuijpers‐Jagtman 2023). As a result, many investigators have attempted to develop interventions and techniques for accelerating tooth movement during orthodontic treatment (Marin et al. 2023; Piroozmand et al. 2022). Previous studies have reported that a number of factors, including hormones (Deng and Guo 2020; Parcianello et al. 2022), nitric oxide (Shirazi et al. 2002), caffeine (Shirazi et al. 2017), ambient pressure (Camacho‐Cardenosa et al. 2019; Shahnavazi, Salari, and Fekrazad 2021), prostaglandins (Illahi et al. 2024), and antidepressants for the management of depression and obsessive‐compulsive disorders (Mirhashemi et al. 2015; Sadrzadeh‐Afshar et al. 2023), may affect OTM and bone metabolism.

Heparin is a sulfated polysaccharide from the glycosaminoglycan family. It has various biological activities, but is widely known for its anticoagulation ability. This medication accelerates the rate at which antithrombin inhibits serine proteases generated during the coagulation cascade. The binding of heparin to antithrombin causes structural modification of antithrombin III and, ultimately, an increased reaction with thrombin (Hogwood et al. 2023). This anti‐anticoagulant agent has been used in the prevention and/or treatment of various issues like venous thromboembolism, embolism in individuals with mechanical heart valves, pregnancy loss in those with anti‐phospholipid antibodies, acute coronary syndrome, atrial fibrillation, and hemofiltration (Ebrahiminik et al. 2021; Gupta, Puttaiahgowda, and Deiglmayr 2024). Its most common side effect is bleeding, but it has also been known to induce thrombocytopenia and osteoporosis (Beurskens et al. 2020). Previous investigations have also reported that heparin could affect bone metabolism. Nishiyama et al. (Wang et al. 2022) reported suppressed bone formation and increased bone resorption following heparin injection in rats and also found that the drug could decrease bone formation markers and cortical thickness. Furthermore, Kanzaki et al. (Kanzaki et al. 2011a) showed that heparin affected bone density and decreased bone mineralization in rats.

Considering the close association between OTM and bone turnover and the results of previous studies indicating a relationship between heparin injection and bone metabolism, we aimed to evaluate the effect of different doses of heparin on OTM in rats and further investigated its possible impact on histomorphometric parameters, bone densitometry, and parathyroid hormone as a bone metabolic marker.

## 2 Materials and Methods

### 2.1 Ethical Statement

The current study was approved by the Ethics Committee of Aja University of Medical Sciences (code no: IR.AJAUMS.REC.1399.112), which operates according to Good Clinical Practice and follows the national guidelines and laws for the care and use of laboratory animals. The experiment procedure and evaluations were conducted at the animal laboratory of Tehran University of Medical Sciences.

### 2.2 Animals and Injections

A total of 24 male, Sprague–Dawley rats (200–250 g) were placed in eight plastic cages and randomly assigned to three groups (*n* = 8) using conventional methods according to the sample size of previous studies and ethical updates (by RM). Groups A, B, and C received subcutaneous injections of 3000 U/kg/d heparin, 6000 U/kg/d heparin (Alborz darou, Tehran, Iran), and 50 U/kg/d normal saline (controls) for 4 weeks, respectively.

The above‐mentioned doses were selected according to general clinical administration of this medicine in previous studies (Qin et al. 2021; Zhang et al. 2023).

Each group of rats was kept in a same cage to minimize potential confounders. All rats were acclimated to a 12‐h light/dark cycle for 1 week before initiation of injections, with free access to water and food. Heparin administrations started from day 1 of the study period and continued for 28 days.

### 2.3 Bone Density Assessment

**Figure 1:**
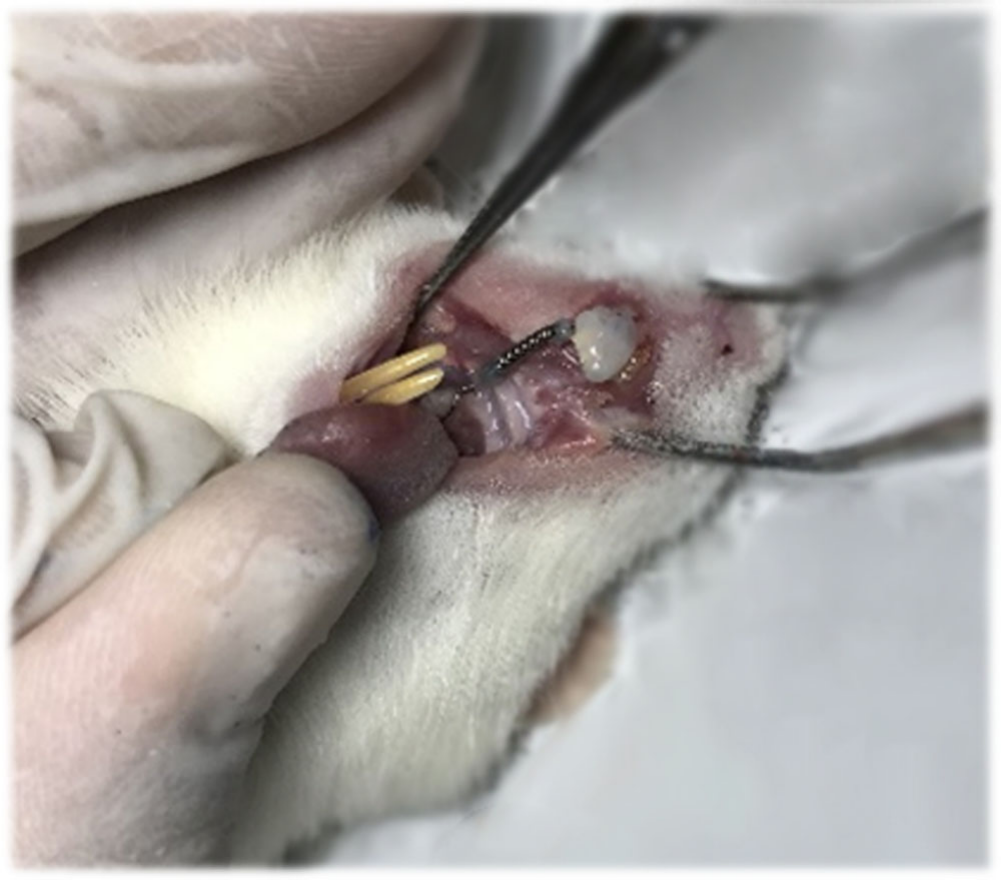
Insertion and activation of the orthodontic appliance between the incisors and the first molar.

Bone mineral density was evaluated by YK through measurement of optical density, bearing in mind that they were inversely correlated (Doustimotlagh et al. 2017). Lateral cephalometric radiographs were obtained by a standard radiographic machine (Trophy, Vincennes, France) using periapical size no 2 (30.5 × 40.5 mm) E‐speed (Carestream, Kodak, Rochester, NY) films. The exposure parameters were 50 kVp and 10 mA, with an exposure time of 0.25 s. The focus‐film distance was constant at 40 cm. Specifications were maintained constant for all animals. Procedures were carried out at baseline and on day 28 under anesthesia using intra‐peritoneal injections of 25 mg/kg ketamine hydrochloride (Rotexmedica, Trittau, Germany) and 8 mg/kg xylazine (Rotexmedica, Trittau, Germany). The radiographic films were processed using an automatic film processor (Velopex Extrax, Medivance, UK). Optical density assessments were performed using a digital densitometer device (TOBIAS TBX‐U, TOBIAS ASSOCIATES, INC IVYLAND, PA) around two points (Figure 1) (Talaeipour et al. 2005):

1 Po point (skull): the most posterior point on the skull.
2 K point (mandible): The intersection between the Gonion and Menton line (Go‐Mn) and perpendicular to the Go‐Mn line through Gnathion (GN).

Comparisons were made between radiographs obtained at baseline and at the end of the study.

### 2.4 Application of Orthodontic Appliances

**Figure 2:**
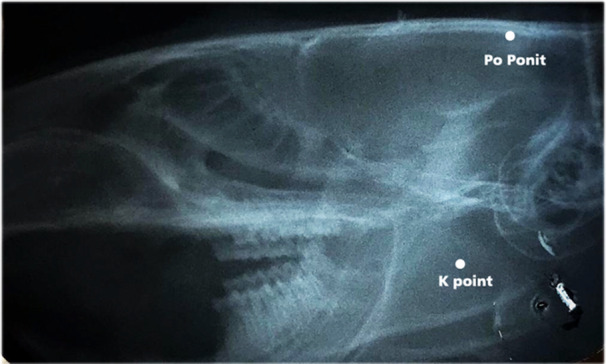
Selected points on the lateral skull radiographs used for optical bone densitometry.

Orthodontic forces were applied 2 weeks after the first injection (day 14), according to previous studies (Shirazi et al. 2017). In brief, each rat was anesthetized by an intra‐peritoneal injection of ketamine hydrochloride (25 mg/kg) and xylazine (8 mg/kg), followed by Ni–Ti closed coil spring (Sentaloy, GAC, NY) insertion between the maxillary right first molar and incisor. This was done using 0.010” ligature wires placed in precut grooves on the labial and distal surfaces of the right incisor. Flowable composite resin was used to cover the wires and to bind the two incisors together in order to provide anchorage for the mesial movement of the molar. Coil springs were activated to deliver a constant force of 60 g (Figure 2). Lower incisor crowns were reduced using a high‐speed handpiece to prevent appliance damage or composite debonding. This was repeated every 4 days until the end of the experiment period. The rats were monitored for loss of reflex, relaxation of muscles, and deep breathing during the procedure and they did not respond to tail or abdominal pinching. The animals were kept warm and vital signs were monitored until full recovery. Standard chow was ground and moistened to ensure appliance retention during eating.

### 2.5 Parathyroid Hormone Measurement

All animals were first weighed and then killed under anesthesia by a sodium pentobarbital overdose on Day 28. Blood samples were drawn directly from the heart after chest incision (and all samples were centrifuged and prepared for hormone measurement using an enzyme‐linked immunosorbent assay (Raybiotech, GA, US).

### 2.6 OTM Measurements

All animals were weighed before they were killed as described above. Following decapitation and before appliance removal, a standard inter‐proximal gauge (Elite, Brassler Co, US) was used to measure the distance between the first and second molars.

### 2.7 Histological Investigation

**Figure 3:**
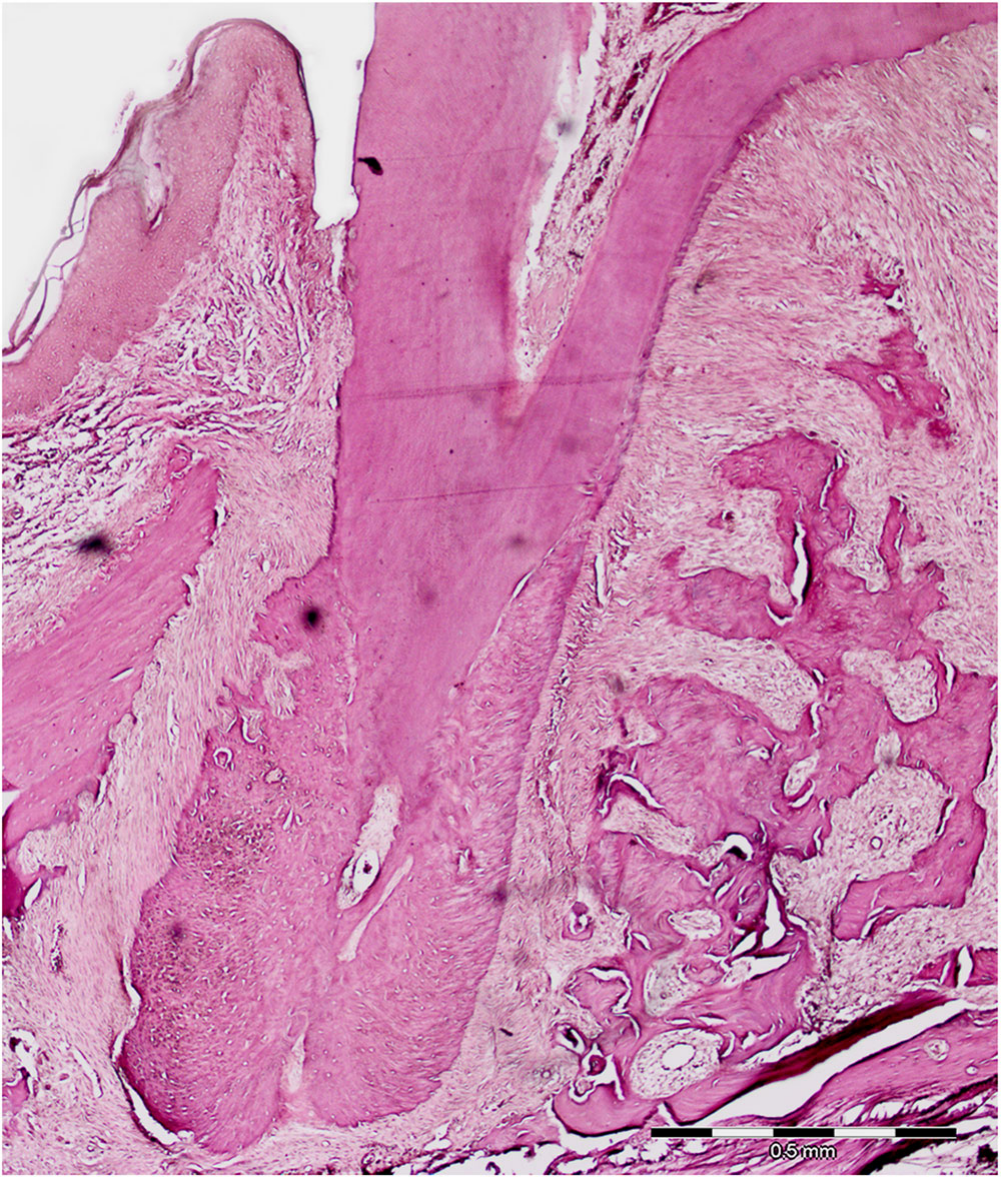
Histopathologic view of the mesiobuccal root of the maxillary first molar used for histologic analysis. (Hematoxylin and Eosin staining; Original magnification ×40).

As described previously (Shirazi et al. 2014, 2017), right hemimaxillae were dissected, fixed in buffered formalin for 48 h, immersed in formic acid until complete decalcification, and routinely processed for histologic evaluation. Five μm‐thick sections were cut from the paraffin‐embedded specimens in a mesio–distal direction and stained with hematoxylin and eosin. Microscopic analysis was performed on the mesiobuccal root of the maxillary first molar (Figure 3) based on previous studies (Sekhavat et al. 2002; Shirazi et al. 2014). Three sections containing the entire root length and the largest root area were selected from each specimen and osteoclast number along with the maximum depth of the resorptive lacunae and lacunae count were determined (Olympus BX51 light microscope, DP25 Olympus digital camera, and DP2‐BSW Olympus analysis software). The mean of the three sections for each measurement was recorded as the final value for that specimen and comparisons were made among groups.

### 2.8 Statistical Analysis

The Kruskal–Wallis test and the Dunn post hoc test were used for statistical analysis by AD. *p* < 0.05 was considered significant.

## 3 Results

All rats survived to the end of the experiment and showed no significant weight loss (*p* > 0.05). The orthodontic appliances remained intact and induced measurable tooth movement in all rats. There was no tooth migration of the left 1st molars in the nontreated sides of the jaws.

### 3.1 Tooth Movement

As demonstrated in Table 1, the highest rate of OTM was found in group B animals receiving 6000 U/Kg/d heparin, whereas the lowest rate of OTM was observed in the controls, indicating a gradual increase in tooth movement with increasing doses of heparin. There was a significant difference in OTM among the three study groups (*p* < 0.001), and based on two‐by‐two comparisons, the rats in group B showed significantly higher OTM compared with the rats in both group A (*p* = 0.005) and the control group (*p* < 0.001).

**Table 1:** Changes in the mean and standard error of mean (SEM) or standard deviation (SD) of the studied variables among the groups.

Groups	OTM (mm) Mean ± SD	PTH (ng/L) Mean ± SD	Osteoclast count (*N*) Mean ± SEM	Lacuna count (*N*) Mean ± SEM	Lacuna depth (µm) Mean ± SEM	Optical density Mean ± SD	
						Skull	Mandible
**Control**	0.41 ± 0.07	5.39 ± 0.82	3.3 ± 1.2	2.5 ± 1	22.76 ± 4.68	1.94 ± 0.09	1.84 ± 0.09
**Heparin 3000 U/Kg**	0.53 ± 0.02	6.08 ± 0.56	4.4 ± 0.08	2 ± 1	30.73 ± 5.16	1.71 ± 0.79	1.54 ± 0.11
**Heparin 6000 U/Kg**	0.75 ± 0.04# ^,^ *	12.18 ± 0.62# ^,^ *	6.4 ± 0.79	4 ± 2# ^,^ *	20.98 ± 0.5	1.98 ± 0.36	1.85 ± 0.41

* *p* < 0.001 among the three groups.

^#^ *p* < 0.05 comparison with group A and controls.

### 3.2 Optical Bone Density

According to our findings shown in Table 1, the optical density of the skull showed no significant difference among the study groups (*p* = 0.12), which was similar to our findings in the mandible (*p* = 0.100).

### 3.3 PTH

The rats in group B showed the highest amount of PTH secretion, followed by the rats in group A and the control group (Table 1). A significant difference was found among the three groups (*p* < 0.001), and two‐by‐two comparisons showed significant differences between Group B with both controls (*p* = 0.001) and Group A (*p* < 0.001). Despite the higher amount of PTH in Group A (3000 U/Kg/d), there was no significant difference between these animals and the control rats (*p* = 0.65).

### 3.4 Histologic Variables

The total number of resorptive lacunae in the mesial aspect of the first molar demonstrated a significant difference among the groups (*p* = 0.03) and was significantly higher in Group B compared with the other groups (both, *p* = 0.04). Neither osteoclast number (*p* = 0.14) nor depth of lacunae (*p* = 0.353) showed significant differences among the study groups.

## 4 Discussion

Tooth movement is directly associated with bone metabolism and turnover. Many medications and external factors could affect this relationship (Eltimamy et al. 2019). Studies on heparin and OTM are limited in the English literature; hence, the current study evaluated the effect of different doses of this medication on OTM and a number of paraclinical factors related to bone metabolism. In this animal study, we applied two common clinical doses of heparin (3000 and 6000 U/kg heparin) to improve the applicability of the results to human clinical scenarios (Zhang et al. 2023). In addition, the force applied to animals’ teeth (60 gram) was selected according to the average force required for tooth movement in humans for the same reason (Y. Li et al. 2021).

Our findings showed that OTM was significantly higher in the group receiving larger doses of heparin (6000 U/Kg/d) compared with those injected with smaller amounts (3000 U/Kg/d) and the controls (saline). In previous studies, it has been reported that bone density is inversely related to OTM (Hsu et al. 2011). Accelerated OTM following heparin administration may be attributed to its ability to decrease bone density and calcification (Salari et al. 2024; Xia et al. 2015). A recent study found a negative impact of heparin on bone density and reported reduced bone absorption markers like alkaline phosphatase and increased resorption factors like pyridinoline following heparin administration in rats. They also suggested that heparin may have an inhibiting effect on calcification due to its high affinity toward calcium ions. We observed that the 3000 U/Kg/d group also had larger OTM values compared with the controls, but the difference was not significant in contrast to its significantly lower OTM compared with the animals receiving 6000 U/Kg/d. This reflects a gradual increase of OTM with increased concentrations (Xia et al. 2015). In agreement with this result, Muir et al. (Muir et al. 1997) used bone turnover markers to assess the effects of heparin on osseous tissues of rats and found a dose‐dependent decrease in cancellous bone volume. Based on our findings, it may be hypothesized that a dose between 3000 U/Kg/d and 6000 U/Kg/d might be the starting point at which the effects of heparin on bone become noticeable. However, this requires further investigation.

According to the results obtained in the present study, PTH increased with increasing doses of heparin and showed significant differences between rats injected with 6000 U/Kg/d and both the other groups (but not between controls and 3000 U/Kg/d), which was in line with our OTM changes. Chen et al. (Chen et al. 2021) demonstrated that PTH regulates calcium and phosphorus levels in extracellular fluid by affecting the kidney and bones. It activates osteoclasts via the RANK–OPG mechanism and decreases bone density to induce an increase in extracellular calcium (Huang et al. 2004). A systematic review summarized that PTH could directly affect the rate and extent of OTM by decreasing bone density (Parcianello et al. 2022). Heparin has been proposed to function as a chelating agent and reduce ionized calcium. This leads to PTH stimulation, ultimately resulting in increased osseous demineralization and osteoclast activity (Xia et al. 2015).

Based on our results, neither the skull nor the mandible showed a significant difference in optical density among the groups, which indicates that the heparin doses used in the 28‐day study period did not induce a significant measurable difference in bone density among the studied rats. Some studies have reported decreased bone density following heparin administration (Douketis et al. 1996). Conversely, it has been suggested that heparin could lead to inhibition of osteoclastic differentiation and function, which hypothetically might lead to an increase in bone density (Wang et al. 2022). Also, a number of clinical trials reported no significant change in bone mineral density following administration of low‐molecular‐weight heparin. These controversial findings might be explained by the binary effect of heparin reported by Kanzaki et al. (Kanzaki et al. 2011b), who suggested that heparin could either activate or suppress osteogenic activity through its different effects on osteoblastic differentiation.

The number of osteoclasts was the highest in the animals in group B, which received the highest dose of heparin (6000 U/Kg/d); however, there were no significant differences among the groups. Alternatively, resorption lacunae showed significantly larger numbers in the 6000 U/Kg/d group compared with both the other groups. These histologic findings support our clinical and biochemical results to some degree. Osteoclasts resorb bone and therefore their increase, especially on the pressure side of the moving roots, can lead to an augmented rate of OTM. Additionally, PTH is known to inhibit the generation and function of osteoprotegerin (OPG), leading to activation of osteoclastogenesis (Irie et al. 2007). An osteoclastogenic effect of heparin has been reported by Li et al (B. Li et al. 2016). Other investigators have indicated that heparin decreased and increased the number/activity of osteoblasts and osteoclasts, respectively (Muir et al. 1997). Osteocyte‐modulated osteoclastogenesis, by inhibition of OPG activity, has also been associated with heparin use (Nozawa et al. 2018). In contrast to these results, Ariyoshi et al. (Ariyoshi et al. 2008) indicated an inhibitory effect for heparin regarding osteoclastogenesis. It is noteworthy that the increase in osteoclast number was not significant, which could be due to an insufficient study sample, or related to limitations in the detection of osteoclasts inherent to hematoxylin/eosin staining compared with more specific staining methods like Tartrate‐resistant acid phosphatase (TRAP). The latter justification is in line with our finding that the number of resorptive lacunae was significantly higher than that in the other groups. On the other hand, the increased bone resorption and OTM rate may be associated with increased osteoclast activity and not osteoclast number, hence the insignificant findings in the current investigation. In addition to the reported effects of heparin and PTH on the activity of osteoclasts discussed above (Huang et al. 2004; Xia et al. 2015), others have also indicated that heparin inhibits OPG activity and therefore activates osteoclastic activity (Irie et al. 2007). The lack of association of lacunar depth with other clinical, biochemical, and histological parameters has also been shown in other studies and requires further investigation (Mirhashemi et al. 2015).

The limitations of the current investigation include a relatively small sample size, a single time interval, and the fact that additional biochemical markers and histologic stains were not used. Future studies with a larger population and sophisticated methods could help to further expand on this subject.

## 5 Conclusions

Heparin increased OTM and PTH in rats and affected histologic bone parameters. This impact seems to be dose‐dependent and becomes significant at doses above 3000 U/Kg/d. Furthermore, heparin reduced optical density of the skull and mandibles.

Orthodontists should consider these effects during treatment planning, especially for those receiving high doses of heparin, such as pregnant women with anti‐phospholipid antibodies and children with hematologic diseases.

## Author Contributions

Behzad Salari, Reza Moradian, and Ahmad Reza Dehpour conceptualized the idea for the research, contributed to the design of the study, literature research, and writing of the manuscript, and carried out proofreading. Yasaman Kheirandish, Shahroo Etemad‐Moghadam, and Mojgan Alaeddini contributed to the design of the study and interpretation of data, and performed the statistical analysis. All authors have contributed to this study and contributed to the paper in equal parts. All authors are in agreement with the content of the manuscript.

## Conflicts of Interest

The authors declare no conflicts of interest.

## Supporting information

Supporting information.

Supporting information.

## Data Availability

The data that support the findings of this study are available on request from the corresponding author. The data are not publicly available due to privacy or ethical restrictions.
