# Supplementary material for: The Effect of Heparin on Bone Metabolism and Orthodontic Tooth Movement in Rats
Source: Clin Exp Dent Res. 2025 Mar 11;11(1):e70061. doi: 10.1002/cre2.70061 (PMC11894433; doi:10.1002/cre2.70061)
Supplement: Supplementary file 2 — Supporting information. [file CRE2-11-e70061-s002.docx]

| ***P-Value*** | **Groups** |  |
| --- | --- | --- |
| 0.005 | B | A |
| 0.201 | C |  |
| 0.005 | A | B |
| 0.005 | C |  |

**OTM S(mm)**

| **Control** | **Heparin 3000 U/Kg** | **Heparin 6000 U/Kg** |
| --- | --- | --- |
| 0.41±0.07 | 0.53±0.02 | 0.75±0.04 |

**Control (C), Heparin 3000 U/Kg (A), Heparin 6000 U/Kg (B)**

**PTH level (ng/L)**

| **Control** | **Heparin 3000 U/Kg** | **Heparin 6000 U/Kg** |
| --- | --- | --- |
| 5.39±0.82 | 6.08±0.56 | 12.18±0.62 |

| ***P-Value*** | **Groups** |  |
| --- | --- | --- |
| 0.000 | B | A |
| 0.787 | C |  |
| 0.001 | C | B |

**Control (C), Heparin 3000 U/Kg (A), Heparin 6000 U/Kg (B)**

**Optical density of skull**

| C | B | A | Group |
| --- | --- | --- | --- |
| 1.75±0.03 | 1.85±0.03 | 1.81±0.01 | Before |
| 1.95±0.04 | 1.98±0.13 | 1.71±0.03 | After |
| 0.06 | 0.43 | 0.04 | *P-Value* |

**Control (C), Heparin 3000 U/Kg (A), Heparin 6000 U/Kg (B)**

**Lacuna count (N)**

| **Control** | **Heparin 3000 U/Kg** | **Heparin 6000 U/Kg** | *P-Value* |
| --- | --- | --- | --- |
| 2.5±1 | 2±1 | 4±2 | 0.03 |

**Control (C), Heparin 3000 U/Kg (A), Heparin 6000 U/Kg (B)**

**Lacuna depth (µm)**

| **Control** | **Heparin 3000 U/Kg** | **Heparin 6000 U/Kg** | *P-Value* |
| --- | --- | --- | --- |
| 22.76±4.68 | 30.73±5.16 | 20.98±0.5 | 0.353 |

**Control (C), Heparin 3000 U/Kg (A), Heparin 6000 U/Kg (B)**

**Osteoclast count (N)**

| **Control** | **Heparin 3000 U/Kg** | **Heparin 6000 U/Kg** | *P-Value* |
| --- | --- | --- | --- |
| 3.3±1.2 | 4.4±0.08 | 6.4±0.79 | 0.131 |

**Control (C), Heparin 3000 U/Kg (A), Heparin 6000 U/Kg (B)**
